# 3-(2-Methyl­phen­yl)-2-thioxo-1,3-thia­zolidin-4-one

**DOI:** 10.1107/S1600536809045814

**Published:** 2009-11-07

**Authors:** Durre Shahwar, M. Nawaz Tahir, Asma Yasmeen, Naeem Ahmad, Muhammad Akmal Khan

**Affiliations:** a Department of Chemistry, Government College University, Lahore, Pakistan; b Department of Physics, University of Sargodha, Sargodha, Pakistan

## Abstract

In the title compound, C_10_H_9_NOS_2_, the 1,3-thia­zolidine and 2-methyl­phenyl rings are oriented at a dihedral angle of 84.44 (9)°. In the crystal, an unusual bifurcated C—H⋯(O,π) inter­action leads to zigzag chains of mol­ecules.

## Related literature

For background to rhodanine derivatives, see: Cutshall *et al.* (2005 ▶). For related structures, see: Shahwar *et al.* (2009*a* ▶,*b* ▶,*c* ▶).
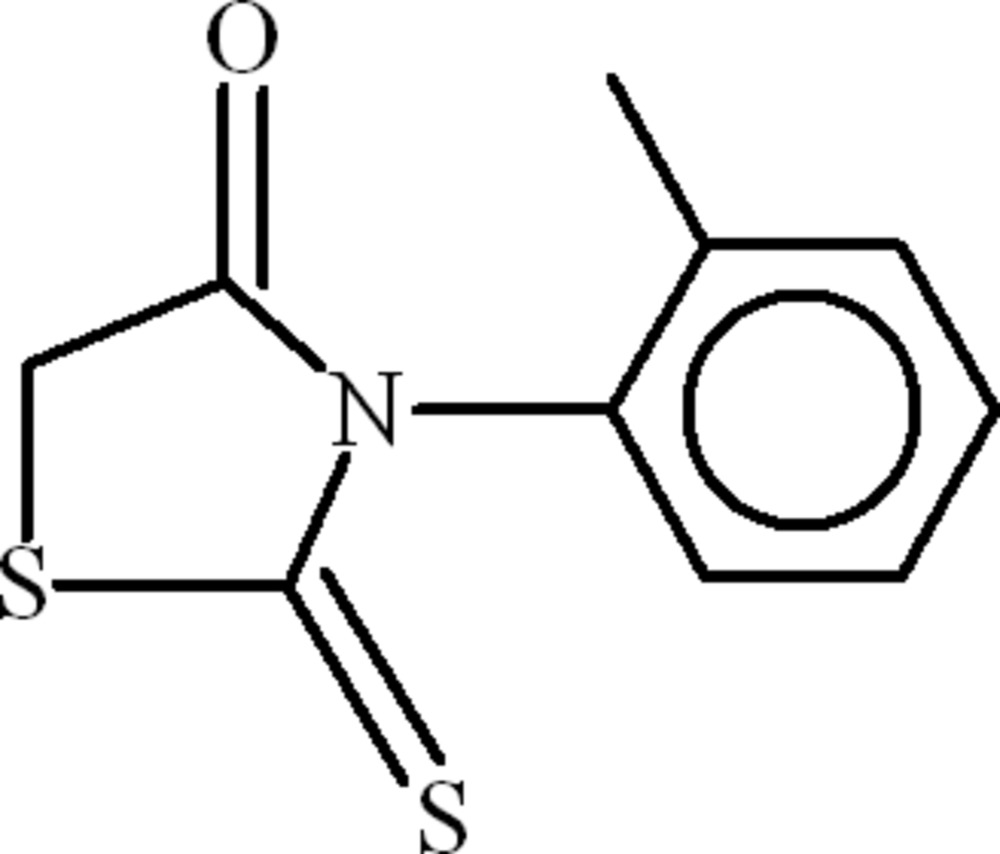


## Experimental

### 

#### Crystal data

C_10_H_9_NOS_2_
*M* *_r_* = 223.30
Monoclinic, 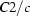

*a* = 23.690 (5) Å
*b* = 7.1401 (17) Å
*c* = 14.628 (3) Å
β = 122.215 (6)°
*V* = 2093.5 (8) Å^3^
*Z* = 8
Mo *K*α radiation
μ = 0.47 mm^−1^
*T* = 296 K
0.34 × 0.16 × 0.14 mm

#### Data collection

Bruker Kappa APEXII CCD diffractometer
Absorption correction: multi-scan (*SADABS*; Bruker, 2005 ▶) *T*_min_ = 0.914, *T*_max_ = 0.934
10878 measured reflections
2661 independent reflections
1436 reflections with *I* > 2σ(*I*)
*R*_int_ = 0.061

#### Refinement

*R*[*F* ^2^ > 2σ(*F* ^2^)] = 0.057
*wR*(*F* ^2^) = 0.189
*S* = 1.02
2661 reflections
128 parameters
H-atom parameters constrained
Δρ_max_ = 0.63 e Å^−3^
Δρ_min_ = −0.31 e Å^−3^

### 

Data collection: *APEX2* (Bruker, 2007 ▶); cell refinement: *SAINT* (Bruker, 2007 ▶); data reduction: *SAINT*; program(s) used to solve structure: *SHELXS97* (Sheldrick, 2008 ▶); program(s) used to refine structure: *SHELXL97* (Sheldrick, 2008 ▶); molecular graphics: *ORTEP-3* (Farrugia, 1997 ▶) and *PLATON* (Spek, 2009 ▶); software used to prepare material for publication: *WinGX* (Farrugia, 1999 ▶) and *PLATON*.

## Supplementary Material

Crystal structure: contains datablocks global, I. DOI: 10.1107/S1600536809045814/hb5205sup1.cif

Structure factors: contains datablocks I. DOI: 10.1107/S1600536809045814/hb5205Isup2.hkl

Additional supplementary materials:  crystallographic information; 3D view; checkCIF report

## Figures and Tables

**Table 1:** Hydrogen-bond geometry (Å, °)

*D*—H⋯*A*	*D*—H	H⋯*A*	*D*⋯*A*	*D*—H⋯*A*
C8—H8*A*⋯O1^i^	0.97	2.58	3.214 (5)	123
C8—H8*A*⋯*Cg*2^i^	0.97	2.65	3.420 (4)	137

Symmetry code: (i) 
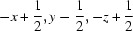
. *Cg*2 is the centroid of the C1–C6 ring.

**Fig. 1.**
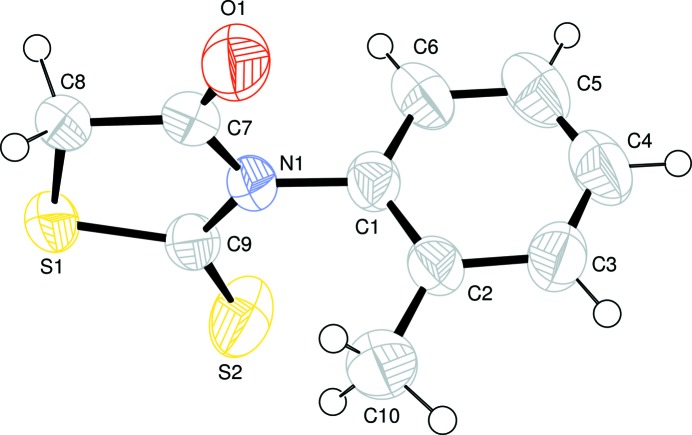
View of (I) with displacement ellipsoids drawn at the 50% probability level.

## supplementary crystallographic information

### Comment

Rhodanine-based molecules have been popular as small molecule inhibitors of numerous targets such as HCV NS3 protease, aldose reductase, beta-lactamase, UDP-*N*-acetylmuramate/*L*-alanine ligase, antidiabetic agents, cathepsin D, and histidine decarboxylase (Cutshall *et al.*, 2005). We herein, report the crystal structure and preparation of the title compound (I, Fig. 1) which is one of the rhodanine derivatives from the series of compounds prepared by our group for beta-lactamase and xanthine oxidase enzyme inhibition studies.

The crystal structures of (II) (5*Z*)-5-(2-Hydroxybenzylidene)-3-phenyl- 2-thioxo-1,3-thiazolidin-4-one (Shahwar *et al.*, 2009*a*), (III) (5*E*)-5-(4-Hydroxy-3-methoxybenzylidene)-2-thioxo-1, 3-thiazolidin-4-one methanol monosolvate (Shahwar *et al.*, 2009*b*) and (IV) (5*Z*)-5-(2-Hydroxybenzylidene)-2-thioxo-1,3-thiazolidin-4-one methanol hemisolvate (Shahwar *et al.*, 2009*c*) have been reported which are the rhodanine derivatives. The crystal stucture of (II) contains (I) as a group.

In (I), the 2-methylphenyl A (C1–C6/C10) and the rhodanine group B (N1/C7/C8/S1/C9/O1/S2) are planar with maximum r. m. s. deviations of 0.0051 and 0.0387 Å respectively, from their mean square planes. The dihedral angle between A/B is 84.44 (9)°. The molecules are stabilized in the form of zig–zag infinte one dimensional polymeric chains due to intermolecular H-bondings (Table 1, Fig. 2). The C–H···π interaction (Table 1) also play a role in stabilizing the molecules.

### Experimental

The title compound was prepared by a three step reaction procedure. In the first step *ortho* toluidine aniline (10.7 g, 0.1 mol) and triethylamine (50.5 g, 0.5 mol) were stirred in ethanol (20 ml) followed by dropwise addition of CS_2_ (15.2 g, 0.2 mol) while keeping the flask in an ice bath. The precipitate obtained were filtered off and washed with diethyl ether.

In second step, a solution of sodium chloroacetate (11.6 g, 0.1 mol) and chloroacetic acid (18.9 g, 0.2 mol) was prepared in 50 ml distilled water. To this solution the precipitates obtained in first step were added gradually and stirred at 273 K. This mixture was stirred untill it turned dark yellow.

In third step the yellow mixture was mixed in 140 ml hot (363–368 K) hydrochloric acid (6 N) and stirred for five minutes to obtain colorless crystalline precipitates. These precipitates were recrystalized in chloroform to get the dark yellow needles of (I).

### Refinement

The coordinates of H2 were refined. The H-atoms were positioned geometrically (C–H = 0.93–0.97 Å) and refined as riding with *U*_iso_(H) = 1.2*U*_eq_(C) or 1.5*U*_eq_(methyl C)..

### Crystal data

**Table:**

C_10_H_9_NOS_2_	*F*(000) = 928
*M**_r_* = 223.30	*D*_x_ = 1.417 Mg m^−^^3^
Monoclinic, *C*2/*c*	Mo *K*α radiation, λ = 0.71073 Å
Hall symbol: -C 2yc	Cell parameters from 2661 reflections
*a* = 23.690 (5) Å	θ = 2.8–28.7°
*b* = 7.1401 (17) Å	µ = 0.47 mm^−^^1^
*c* = 14.628 (3) Å	*T* = 296 K
β = 122.215 (6)°	Cut needle, dark yellow
*V* = 2093.5 (8) Å^3^	0.34 × 0.16 × 0.14 mm
*Z* = 8	

### Data collection

**Table:**

Bruker Kappa APEXII CCD diffractometer	2661 independent reflections
Radiation source: fine-focus sealed tube	1436 reflections with *I* > 2σ(*I*)
graphite	*R*_int_ = 0.061
Detector resolution: 7.40 pixels mm^-1^	θ_max_ = 28.7°, θ_min_ = 2.8°
ω scans	*h* = −31→30
Absorption correction: multi-scan (*SADABS*; Bruker, 2005)	*k* = −9→5
*T*_min_ = 0.914, *T*_max_ = 0.934	*l* = −17→19
10878 measured reflections	

### Refinement

**Table:**

Refinement on *F*^2^	Primary atom site location: structure-invariant direct methods
Least-squares matrix: full	Secondary atom site location: difference Fourier map
*R*[*F*^2^ > 2σ(*F*^2^)] = 0.057	Hydrogen site location: inferred from neighbouring sites
*wR*(*F*^2^) = 0.189	H-atom parameters constrained
*S* = 1.02	*w* = 1/[σ^2^(*F*_o_^2^) + (0.0949*P*)^2^ + 0.6816*P*] where *P* = (*F*_o_^2^ + 2*F*_c_^2^)/3
2661 reflections	(Δ/σ)_max_ < 0.001
128 parameters	Δρ_max_ = 0.63 e Å^−^^3^
0 restraints	Δρ_min_ = −0.31 e Å^−^^3^

### Special details

**Table:**

Geometry. Bond distances, angles *etc*. have been calculated using the rounded fractional coordinates. All su's are estimated from the variances of the (full) variance-covariance matrix. The cell e.s.d.'s are taken into account in the estimation of distances, angles and torsion angles
Refinement. Refinement of *F*^2^ against ALL reflections. The weighted *R*-factor *wR* and goodness of fit *S* are based on *F*^2^, conventional *R*-factors *R* are based on *F*, with *F* set to zero for negative *F*^2^. The threshold expression of *F*^2^ > σ(*F*^2^) is used only for calculating *R*-factors(gt) *etc*. and is not relevant to the choice of reflections for refinement. *R*-factors based on *F*^2^ are statistically about twice as large as those based on *F*, and *R*- factors based on ALL data will be even larger.

### Fractional atomic coordinates and isotropic or equivalent isotropic displacement parameters (Å^2^)

**Table:**

	*x*	*y*	*z*	*U*_iso_*/*U*_eq_	
S1	0.21396 (5)	−0.00494 (13)	−0.00417 (7)	0.0612 (3)	
S2	0.07182 (5)	0.09169 (18)	−0.10373 (8)	0.0903 (4)	
O1	0.26644 (11)	0.2834 (4)	0.24779 (19)	0.0733 (9)	
N1	0.17183 (11)	0.1968 (3)	0.09176 (18)	0.0475 (8)	
C1	0.12842 (14)	0.2941 (4)	0.1167 (2)	0.0499 (10)	
C2	0.11018 (15)	0.4752 (5)	0.0824 (2)	0.0527 (10)	
C3	0.06977 (17)	0.5655 (6)	0.1131 (3)	0.0672 (12)	
C4	0.05067 (19)	0.4719 (7)	0.1737 (3)	0.0768 (16)	
C5	0.0691 (2)	0.2946 (7)	0.2068 (3)	0.0779 (16)	
C6	0.10811 (17)	0.2011 (6)	0.1777 (3)	0.0660 (14)	
C7	0.23984 (15)	0.1956 (5)	0.1655 (2)	0.0509 (11)	
C8	0.27516 (16)	0.0721 (5)	0.1295 (3)	0.0555 (11)	
C9	0.14858 (16)	0.1022 (4)	−0.0033 (3)	0.0537 (11)	
C10	0.1303 (2)	0.5694 (6)	0.0175 (3)	0.0726 (14)	
H3	0.05615	0.68853	0.09201	0.0808*	
H4	0.02385	0.53341	0.19283	0.0918*	
H5	0.05568	0.23555	0.24881	0.0933*	
H6	0.12066	0.07745	0.19869	0.0793*	
H8A	0.29480	−0.03430	0.17767	0.0668*	
H8B	0.31039	0.14084	0.12918	0.0668*	
H10A	0.11483	0.50009	−0.04790	0.1090*	
H10B	0.17806	0.57808	0.05699	0.1090*	
H10C	0.11140	0.69291	0.00017	0.1090*	

### Atomic displacement parameters (Å^2^)

**Table:**

	*U*^11^	*U*^22^	*U*^33^	*U*^12^	*U*^13^	*U*^23^
S1	0.0782 (6)	0.0598 (6)	0.0604 (5)	0.0158 (4)	0.0468 (5)	0.0023 (4)
S2	0.0723 (7)	0.0974 (9)	0.0678 (7)	0.0173 (6)	0.0150 (5)	−0.0333 (6)
O1	0.0542 (14)	0.097 (2)	0.0562 (14)	0.0095 (13)	0.0211 (12)	−0.0126 (14)
N1	0.0499 (14)	0.0541 (16)	0.0422 (13)	0.0081 (11)	0.0271 (12)	−0.0011 (11)
C1	0.0464 (16)	0.0565 (19)	0.0440 (16)	0.0030 (13)	0.0222 (14)	−0.0077 (14)
C2	0.0533 (18)	0.064 (2)	0.0423 (16)	0.0056 (14)	0.0265 (15)	−0.0023 (14)
C3	0.060 (2)	0.075 (2)	0.057 (2)	0.0178 (17)	0.0247 (17)	−0.0056 (18)
C4	0.057 (2)	0.121 (4)	0.061 (2)	0.002 (2)	0.0373 (19)	−0.013 (2)
C5	0.076 (3)	0.103 (3)	0.074 (2)	−0.016 (2)	0.053 (2)	−0.012 (2)
C6	0.066 (2)	0.086 (3)	0.0577 (19)	−0.0094 (18)	0.0409 (18)	−0.0094 (18)
C7	0.0538 (18)	0.059 (2)	0.0442 (17)	0.0084 (15)	0.0291 (15)	0.0084 (15)
C8	0.0607 (19)	0.063 (2)	0.0565 (18)	0.0147 (15)	0.0404 (17)	0.0164 (16)
C9	0.069 (2)	0.0464 (18)	0.0475 (17)	0.0080 (14)	0.0323 (16)	−0.0041 (14)
C10	0.089 (3)	0.062 (2)	0.079 (2)	0.0073 (19)	0.053 (2)	0.008 (2)

### Geometric parameters (Å, °)

**Table:**

S1—C8	1.790 (4)	C4—C5	1.343 (7)
S1—C9	1.734 (4)	C5—C6	1.379 (7)
S2—C9	1.619 (4)	C7—C8	1.492 (6)
O1—C7	1.196 (4)	C3—H3	0.9300
N1—C1	1.440 (4)	C4—H4	0.9300
N1—C7	1.381 (4)	C5—H5	0.9300
N1—C9	1.370 (4)	C6—H6	0.9300
C1—C2	1.371 (5)	C8—H8A	0.9700
C1—C6	1.388 (5)	C8—H8B	0.9700
C2—C3	1.412 (6)	C10—H10A	0.9600
C2—C10	1.436 (6)	C10—H10B	0.9600
C3—C4	1.366 (6)	C10—H10C	0.9600
			
C8—S1—C9	93.61 (19)	S2—C9—N1	126.4 (3)
C1—N1—C7	119.9 (2)	C2—C3—H3	120.00
C1—N1—C9	122.7 (3)	C4—C3—H3	120.00
C7—N1—C9	117.4 (3)	C3—C4—H4	119.00
N1—C1—C2	119.4 (3)	C5—C4—H4	119.00
N1—C1—C6	118.1 (3)	C4—C5—H5	120.00
C2—C1—C6	122.5 (3)	C6—C5—H5	120.00
C1—C2—C3	116.7 (3)	C1—C6—H6	120.00
C1—C2—C10	122.2 (4)	C5—C6—H6	120.00
C3—C2—C10	121.1 (4)	S1—C8—H8A	110.00
C2—C3—C4	119.9 (4)	S1—C8—H8B	110.00
C3—C4—C5	122.6 (5)	C7—C8—H8A	110.00
C4—C5—C6	119.2 (4)	C7—C8—H8B	110.00
C1—C6—C5	119.1 (4)	H8A—C8—H8B	109.00
O1—C7—N1	123.5 (3)	C2—C10—H10A	109.00
O1—C7—C8	124.9 (3)	C2—C10—H10B	109.00
N1—C7—C8	111.6 (3)	C2—C10—H10C	109.00
S1—C8—C7	106.7 (3)	H10A—C10—H10B	110.00
S1—C9—S2	123.3 (2)	H10A—C10—H10C	110.00
S1—C9—N1	110.4 (3)	H10B—C10—H10C	110.00
			
C9—S1—C8—C7	−4.5 (3)	C7—N1—C9—S2	−177.0 (3)
C8—S1—C9—S2	−179.4 (2)	N1—C1—C2—C3	−177.5 (3)
C8—S1—C9—N1	1.8 (3)	N1—C1—C2—C10	3.1 (4)
C7—N1—C1—C2	94.3 (3)	C6—C1—C2—C3	0.6 (5)
C7—N1—C1—C6	−84.0 (4)	C6—C1—C2—C10	−178.8 (3)
C9—N1—C1—C2	−86.2 (4)	N1—C1—C6—C5	177.0 (3)
C9—N1—C1—C6	95.6 (4)	C2—C1—C6—C5	−1.2 (5)
C1—N1—C7—O1	−5.7 (5)	C1—C2—C3—C4	−0.2 (5)
C1—N1—C7—C8	174.3 (3)	C10—C2—C3—C4	179.2 (4)
C9—N1—C7—O1	174.7 (3)	C2—C3—C4—C5	0.4 (6)
C9—N1—C7—C8	−5.4 (4)	C3—C4—C5—C6	−0.9 (7)
C1—N1—C9—S1	−177.9 (2)	C4—C5—C6—C1	1.3 (6)
C1—N1—C9—S2	3.4 (4)	O1—C7—C8—S1	−173.9 (3)
C7—N1—C9—S1	1.8 (3)	N1—C7—C8—S1	6.1 (4)

### Hydrogen-bond geometry (Å, °)

**Table:**

*D*—H···*A*	*D*—H	H···*A*	*D*···*A*	*D*—H···*A*
C8—H8A···O1^i^	0.97	2.58	3.214 (5)	123
C8—H8A···Cg2^i^	0.97	2.65	3.420 (4)	137

Symmetry codes: (i) −*x*+1/2, *y*−1/2, −*z*+1/2.
